# How Prevalent Is Schizophrenia?

**DOI:** 10.1371/journal.pmed.0020146

**Published:** 2005-05-31

Schizophrenia is a devastating mental illness and a major contributor to the global burden of disease. In their quest to understand schizophrenia epidemiology, John McGrath and colleagues have previously undertaken a systematic review of schizophrenia incidence—that is, the number of new cases diagnosed each year in a specified population (see *BMC Medicine* 2: e13). They now report results from a second systematic review that examines published studies on the prevalence of the disease—i.e., on the number of people who are suffering from the disease at a given time or within a specified time interval. (Incidence studies can suggest risk factors that may underlie variations in the disease. Prevalence studies are central to health systems planning.)

Analyzing a total of 1,721 estimates from 188 studies and covering 46 countries, they calculated the following median prevalence estimates: 4.6 per 1,000 for point prevalence (defined as prevalence during any interval of less than a month), 3.3 for period prevalence (defined as prevalence during a period from 1 to 12 months), 4.0 for lifetime prevalence (the proportion of individuals in the population who have ever manifested the disease and who are alive on a given day), and 7.2 for lifetime morbid risk (which attempts to include the entire lifetime of a birth cohort, both past and future, and includes those deceased at the time of the survey).

These numbers are consistent with key policy documents about point prevalence, but suggest that the 0.5%–1% estimate for lifetime prevalence given in many textbooks is an overestimate. This estimate, the authors suggest, “is another example where the research community needs to review their belief systems in the face of data.” Another often quoted statistic, namely that “schizophrenia affects about one in a hundred” most sensibly refers to lifetime morbid risk data. Here as well, the systematic analysis suggests that the reality is somewhat lower, and the authors suggest that “if we wish to provide the general public with a measure of the likelihood that individuals will develop schizophrenia during their lifetime, then a more accurate statement would be that about seven to eight individuals per 1,000 will be affected.”

The authors were surprised to find no difference in prevalence between males and females, because their incidence review had found a male/female risk ratio of 1.4. On the other hand, the incidence study had revealed a higher incidence among migrant groups than among native-born individuals, and this was true for prevalence estimates as well. Compared to economically developed nations, the prevalence of schizophrenia is lower in developing nations, which is consistent with the literature showing that the course (i.e., prognosis) of schizophrenia is better in developing nations.

Systematic reviews are secondary research, where the object of scrutiny is not the prevalence of schizophrenia per se but the literature on the topic, and the estimates in this review have to be treated accordingly. Regardless of exact numbers, however, the authors conclude that “many people with schizophrenia have persisting symptoms, despite the best mix of interventions we can offer.” It has been estimated that current interventions can at most reduce 25% of disease burden, thus the authors conclude that “this is a powerful argument for investing in applied and basic research.”

