# Correction: How Prevalent Is Schizophrenia?

**DOI:** 10.1371/journal.pmed.1002925

**Published:** 2019-08-30

**Authors:** The PLOS Medicine Staff

## Notice of Republication

This article was republished on August 15, 2019, to remove the article figure and its accompanying legend from publication by request of the original artist due to a change in copyright.
